# Supplementary material for: Modeling glioblastoma heterogeneity as a dynamic network of cell states
Source: Mol Syst Biol. 2021 Sep 16;17(9):e10105. doi: 10.15252/msb.202010105 (PMC8444284; doi:10.15252/msb.202010105)
Supplement: Supplementary file 6 — Source Data for Figure 5 [file MSB-17-e10105-s004.zip › Figure5A_sourcedata/GSEA_3071/nosigdb_stateVI.GseaPreranked.1622795169056/RICHARDS_LIDDELOW_NATURE_2017_A2_REACTIVE_ASTROCYTES.html]

Details for gene set RICHARDS\_LIDDELOW\_NATURE\_2017\_A2\_REACTIVE\_ASTROCYTES[GSEA]

|  || Dataset | state63071 |
| Phenotype | NoPhenotypeAvailable |
| Upregulated in class | na\_pos |
| GeneSet | RICHARDS\_LIDDELOW\_NATURE\_2017\_A2\_REACTIVE\_ASTROCYTES |
| Enrichment Score (ES) | 0.43123916 |
| Normalized Enrichment Score (NES) | 1.9282346 |
| Nominal p-value | 0.007168459 |
| FDR q-value | 0.008950633 |
| FWER p-Value | 0.077 |
Table: GSEA Results Summary

  

Fig 1: Enrichment plot: RICHARDS\_LIDDELOW\_NATURE\_2017\_A2\_REACTIVE\_ASTROCYTES      
 Profile of the Running ES Score & Positions of GeneSet Members on the Rank Ordered List

  

| PROBE | GENE SYMBOL | GENE\_TITLE | RANK IN GENE LIST | RANK METRIC SCORE | RUNNING ES | CORE ENRICHMENT || 1 | HMGA1 |  |  | 1 | 1.500 | 0.0977 | Yes |
| 2 | PTX3 |  |  | 56 | 0.860 | 0.1389 | Yes |
| 3 | ANXA2 |  |  | 79 | 0.783 | 0.1840 | Yes |
| 4 | GADD45B |  |  | 90 | 0.758 | 0.2307 | Yes |
| 5 | AKAP12 |  |  | 92 | 0.743 | 0.2790 | Yes |
| 6 | TM4SF1 |  |  | 123 | 0.703 | 0.3166 | Yes |
| 7 | FLNC |  |  | 134 | 0.685 | 0.3586 | Yes |
| 8 | SPHK1 |  |  | 141 | 0.677 | 0.4011 | Yes |
| 9 | KLF5 |  |  | 282 | 0.548 | 0.3982 | Yes |
| 10 | HSPB1 |  |  | 322 | 0.527 | 0.4218 | Yes |
| 11 | CTGF |  |  | 404 | 0.487 | 0.4312 | Yes |
| 12 | TUBB6 |  |  | 539 | 0.444 | 0.4232 | No |
| 13 | VIM |  |  | 897 | 0.361 | 0.3482 | No |
| 14 | B3GNT5 |  |  | 1308 | 0.302 | 0.2546 | No |
| 15 | CLCF1 |  |  | 1592 | 0.269 | 0.1940 | No |
| 16 | GCNT2 |  |  | 1598 | 0.269 | 0.2102 | No |
| 17 | GAP43 |  |  | 3568 | -1.438 | -0.2398 | No |
| 18 | IGFBP3 |  |  | 3588 | -1.623 | -0.1391 | No |
| 19 | GFAP |  |  | 3619 | -2.337 | 0.0052 | No |
Table: GSEA details [plain text format]

  

Fig 2: RICHARDS\_LIDDELOW\_NATURE\_2017\_A2\_REACTIVE\_ASTROCYTES: Random ES distribution      
 Gene set null distribution of ES for **RICHARDS\_LIDDELOW\_NATURE\_2017\_A2\_REACTIVE\_ASTROCYTES**
